# Impact of seasonality and forest stand age on ion deposition in rehabilitated forests

**DOI:** 10.1002/pei3.70005

**Published:** 2024-08-20

**Authors:** Mohamad Hilmi Ibrahim, Salwana Jaafar, Naoyuki Yamashita, Hiroyuki Sase

**Affiliations:** ^1^ Agrotechnology Programme, Faculty of Resources Science and Technology Universiti Malaysia Sarawak Kota Samarahan Sarawak Malaysia; ^2^ Institute for Biodiversity and Environmental Research, Universiti Brunei Darussalam Gadong Brunei Darussalam; ^3^ Forestry and Forest Products Research Institute Tsukuba Ibaraki Japan; ^4^ Asia Center for Air Pollution Research (ACAP) Japan Environmental Sanitation Center Niigata‐shi Japan

**Keywords:** foliar leaching, internal precipitation, ion‐exchange resin sampler, nutrient cycling, through‐fall

## Abstract

This study examines the critical interaction between seasonal precipitation variability and forest maturity in determining ion deposition patterns in rehabilitated forest ecosystems. This research was conducted in rehabilitated forest sites in Bintulu, Sarawak, Malaysia that had ecologically similar plant distribution, species, and age in each planting area. This facilitated the standardization of rainfall deposition in the different study plots which streamlined the study of these specific facets of ecosystem dynamics. The goal is to understand how seasonal changes and the age of the forest influence the chemical composition of the flux that relates to the movement and deposition of nutrients through the forest ecosystem. This flux is a key factor in the health of the forest ecosystem and nutrient cycling. Using ion exchange resin (IER) samplers, we accurately measured and compared the deposition of different ions (Ca^2+^, Na^+^, Fe^2+^, Cu^2+^, NO_3_^−^, NH_4_^+^ and SO_4_^2−^) across different seasons and forest ages. The deposition of Ca^2+^ and NH₄^+^ was significantly lower in the low‐precipitation season than in the high‐precipitation season in all forest stands, regardless of the year they were established (1996, 1999, 2002, 2005, and 2009). In contrast, ions such as Na^+^, Fe^2+^, Cu^2+^, NO_3_^−^ and SO_4_^2−^ showed no clear seasonal fluctuations. In addition, the study shows that through‐fall in forest stands from 2002, 2005 and 2009 had higher concentrations of Ca^2+^ in both seasons than in 1996 and 1999. Interestingly, forest stands from 2009 and 2002 had elevated levels of Na^+^ and SO₄^2−^ in seasons with low precipitation, while stands from 1996 had higher levels in seasons with high precipitation. Our results emphasize the crucial role of precipitation amount and canopy age in determining ion deposition in forest ecosystems. By demonstrating the significant influence of precipitation seasonality and forest maturity on the chemical composition of throughfall, this study contributes to a deeper understanding of nutrient dynamics in developing forest landscapes and provides valuable insights for ecological restoration measures.

## 1 INTRODUCTION

Dry deposition refers to the process by which gaseous and particulate pollutants are transferred directly from the atmosphere to surfaces such as vegetation, water bodies or soil without precipitation. Wet deposition, on the other hand, is the process in which pollutants are washed out of the atmosphere by precipitation such as rain, snow, fog or dew. Wet deposition serves as an input into the ecosystem (Mladenov et al., 2012; Talkner et al., 2010) provides important nutrients in oligotrophic habitats (Hämmerle et al., 2018) and introduces pollutants (Igawa et al., 2002). According to the reports of Zhang et al. (2007) and Huang et al. (2009) in southern China, wet deposition accumulates about 94 kg hectare^−1^ of nutrient deposition, while dry deposition accounts for only 6 kg hectare^−1^ of nutrient deposition. Wet deposition is also called acid rain or acid deposition when the concentrations of sulfur dioxide (SO₂) and nitrogen oxides (NOx) in rainwater exceed 0.254 and 0.752 ppb, respectively (US‐EPA, 2005). Therefore, both wet and dry deposition, or seasonal deposition, is crucial to identify critical periods for nutrient cycling, forest health, and the influence of weather patterns on forest health and deposition rates (Liu et al., 2008), and to track the influence of seasonal weather patterns on deposition rates.

As Hicks et al. (2016) emphasize, the process of dry deposition in the tree canopy plays a crucial role in trapping gaseous and particulate matter during dry periods. This process is followed by the wet deposition of these substances by rainwater, as Tan et al. (2018) show that the importance of this phenomenon lies in its contribution to the development of a seasonal signal in deposition. This is supported by the study of Rummel et al. (2007), which showed that the average deposition velocity at midday in the dry burning season was only 0.5 cm s^−1^, indicating a significant influence of dry periods on deposition. In addition, the work of Cao et al. (2006) emphasized the role of fog and water deposition in alleviating water stress in plants during the dry season, further underlining the importance of wet deposition after dry periods.

During a dry period, the forest canopy can trap gaseous and particulate matter through the process of dry deposition (Hicks et al., 2016). After exchange processes in the forest canopy, such dry‐deposited substances are washed out by rainwater on a rainy day (wet deposition) (Tan et al., 2018). The ions are then absorbed or washed out by the diffusion mechanism of the tree. During absorption, the ions are exchanged with the leaf tissue through the stomata and cuticular layer, and during leaching, they are flushed directly to the forest floor by the throughfall and stemflow (Gessler et al., 2002; Sase et al., 2008; Talkner et al., 2010). This process is influenced by plant physiology, such as waxing, cuticle, apoplast, and xylem sap (Hambuckers & Remacle, 1993). As a result, tree canopies can intercept harmful pollutants such as SO_4_^2−^ and NO_3_^−^ from acid precipitation or acid rain and remove large amounts of air pollution, thereby improving air quality in cities. However, by intercepting and accumulating these pollutants, tree canopies can also exacerbate the negative effects of acid rain.

In a rehabilitated forest, recent studies have revealed intriguing links between ecological restoration processes and the effects of ion deposition. Studies have shown that an increase in ion deposition, e.g. of nitrogen and sulfur compounds, can alter soil chemistry, leading to changes in nutrient availability, soil pH, and overall soil fertility. Consequently, these changes can affect the composition and diversity of plant communities. In addition, the effects of ion deposition on microbial communities, which play a critical role in nutrient cycling and the overall functioning of restored and rehabilitated forests, should also be considered. These findings emphasize the importance of understanding the complex interactions between forest restoration and ion input, as they play a critical role in shaping the resilience and ecological success of restored forest landscapes. To date, little research has been conducted on nutrient inputs to restored forests through atmospheric deposition, particularly the effects of ion deposition during different precipitation periods and pollutant uptake by tree canopies. This study aims to fill these gaps by investigating how different precipitation patterns influence ion deposition and how tree canopies contribute to the absorption and attenuation of atmospheric pollutants, providing crucial insights into the nutrient dynamics and ecological health of restored forests.

Malaysia's cumulative acid load from the atmosphere for terrestrial ecosystems has increased from 2010 to 2020 (EANET (Acid Deposition Monitoring Network in East Asia), 2021a). In 2020, wet deposition levels of SO_4_^2−^, NO_3_^−^ and NH_4_^+^ outside the sea were recorded at the Petaling Jaya sites, which were above the 80th percentile of EANET sites (EANET (Acid Deposition Monitoring Network in East Asia), 2021b). Tropical tree canopy structure and organization, such as branch angle, canopy depth, canopy cover, and leaf area index, influence rainwater infiltration and nutrient cycling in forests (Crockford & Richardson, 2000; Park & Cameron, 2008). Heavy rainfall can affect the solubility of rainwater, which has an impact on nutrient cycling in forests (Khormali et al., 2009).

With this in mind, we sought to investigate the differences in ionic deposition for Ca^2+^, Na^+^, Fe^2+^, Cu^2+^, NH_4_^+^, NO_3_^−^ and SO_4_^2−^ in the through‐fall of a rehabilitated forest at Universiti Putra Malaysia Bintulu Campus (UPMKB) in Sarawak, Malaysia. We hypothesized that different precipitation seasons and forest stands would have different effects on through‐fall deposition of Ca^2+^, Na^+^, Fe^2+^, Cu^2+^, NH_4_^+^, NO_3_^−^, and SO_4_^2−^ ions in forest sites. By investigating ion deposition in a rehabilitated forest on the campus of Universiti Putra Malaysia Bintulu in Sarawak, the study shows the influence of different rainfall periods. This research fills a knowledge gap by investigating the interactions between the dynamics of rehabilitated forests and seasonal variations, which are not extensively addressed in the existing literature. The key questions addressed in the study are as follows:

1. How does the precipitation season influence deposition trends across different types of forest stands?
2. Does the state of ion deposition in rehabilitated forests depend on the forest stands?

## 2 METHODS

### 2.1 Study sites

**FIGURE 1:**
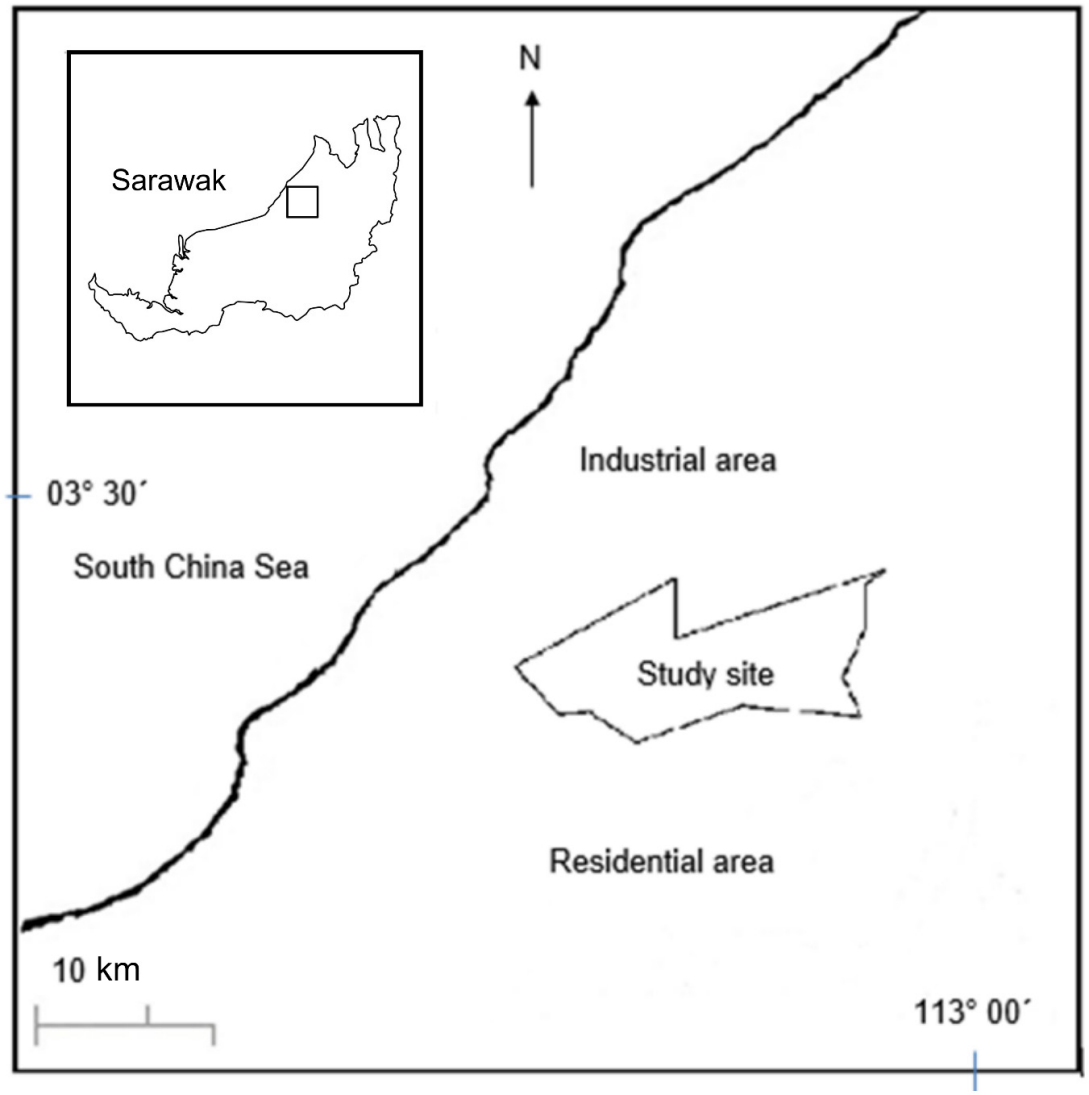
Location of the ion deposition study in rehabilitated forest UPMKB, Bintulu, Sarawak. Through‐fall monitoring was conducted in fifteen 20 × 20 m plots in study sites (*n* = 3) adapted from Ibrahim et al. (2021).

The study was conducted over 1 year in 2011 in a rehabilitated forest on the Universiti Putra Malaysia Bintulu (UPMKB) campus in Sarawak (Figure 1). The rehabilitated forests at UPMKB have similar plant distribution, species and age in each planting area, which facilitated the standardization of rainfall deposition across the different study sites. This approach allowed for a more comprehensive understanding of ecosystem responses to restoration activities.

The forest, which was initiated in collaboration with Mitsubishi Corporation, is a thriving ecosystem in Bintulu, Sarawak, Malaysia. Reforestation efforts began in the 1990s with the aim of restoring the degraded forest area around the campus to its original state. Native tree species such as Dipterocarpaceae, Myrtaceae, Anarcadiaceae, and Guttiferae were planted, and sustainable forestry practices were introduced to ensure the long‐term viability of the forest.

The rehabilitated forest was divided into five forest stands, each corresponding to the years of their establishment (1996, 1999, 2002, 2005, and 2009), with the overall topography consisting of small hills. The average tree height of each forest stand was 9.30 ± 0.24, 6.15 ± 0.13, 4.02 ± 0.14, 2.43 ± 0.11, and 0.46 ± 0.15 m, respectively, while the average basal area per hectare of each forest stand was 0.56, 0.80, 0.64, 0.37 and 0.02 m^2^/ha, respectively (Heng et al., 2011). Heng et al. (2017) also measured the canopy openness of each forest stand, which ranged from 6% to 23% for 2009, 30% to 35% for 2005, 51% to 59% for 2002, 56% to 61% for 1999, and 77% to 79% for 1996 respectively. Based on the canopy openness results, forest stands with an openness of more than 50% were categorized as open stands. Consequently, the forest stands established in 1996, 1999, and 2002 were classified as dense stands, while the stands established in 2005 and 2009 were classified as open stands. In all five forest stands, the dominant tree family is the Dipterocarpaceae with a total of 56 species. After this family, Myrtaceae is the second most abundant family with a total of 9 species. Anarcadiacea and Guttiferae are also represented with 7 species each. In the forest rehabilitated by UPMKB, a total of 15 plots of 20 × 20 m were established: 3 plots (with 10 m distance between the plots) in each forest stand, in 1996, 1999, 2002, 2005 and 2009 (Table 1).

**TABLE 1:** Description of different years of forest stand establishments (1996, 1999, 2002, 2005 and 2009) in UPMKB rehabilitation forest (*n* = 3 plots per site). The tree's mean height, mean basal area in 0.04 h^−1^ and canopy openness based on Heng et al. (2011, 2017).

Forest stand (year)	Location	Mean tree height (m)	Mean basal area value in hectare	Canopy openness (%)	Plot classification
2009	03°12.721′N, 113°03.765′E	0.46 ± 0.15	0.02	6–23	Open stand
2005	03°12.834′N, 113°04.456′E	2.43 ± 0.11	0.37	30–35	Open stand
2002	03°12.735′N, 113°04.492′E	4.02 ± 0.14	0.64	51–59	Dense stand
1999	03°12.727′N, 113°04.492′E	6.15 ± 0.13	0.80	56–61	Dense stand
1996	03°12.768′N, 113°04.470′E	9.30 ± 0.24	0.56	77–79	Dense stand

### 2.2 Environmental conditions in the study area

During the 12‐month study period in 2011, a total of 67 rainy days were observed, spread over 4 months, which corresponds to a total rainfall of around 1088 mm. The monthly average temperature was measured at Bintulu International Airport, which is about 14 km away from the study area (Malaysian Meteorological Department (MMD, 2011). Each forest stand was equipped with a thermometer (MAA‐10B, Gilson Company Inc., USA) and a loop hygrometer to measure temperature and relative humidity (116A, Elcometer Inc., UK). In each forest stand, environmental parameters (air temperature and relative humidity) varied between 28.3 and 30.7°C and humidity between 72% and 86%.

The rainy and dry seasons in tropical countries such as Malaysia are determined by different climatic patterns such as the monsoon and trade winds. The rainy season, which is characterized by heavy rainfall, usually lasts from May to October, while the dry season with low rainfall extends from November to April (Guo, Hu, & Guan, 2022; Guo, Yan, et al., 2022). These seasons are determined by factors such as evapotranspiration, geopotential height and atmospheric circulation (Qian & Tang, 2010; Xu et al., 2022). Based on the above statement, we classify the seasons into high‐precipitation and low‐precipitation periods. Accordingly, we conducted our sampling during these different periods. The high rainfall season, or rainy season, from May to October, is ideal for observing conditions during heavy rainfall. In contrast, the low rainfall, or dry season, from November to April, provides the opportunity to study conditions during low rainfall. This approach ensures comprehensive data collection under different climatic conditions.

### 2.3 Ion exchange resin (IER) sampler for ion deposition via through‐fall

The IER sampler method described by Ibrahim et al. (2021) was used to quantify the deposition of cations and anions from the atmosphere to the forest floor. Each sampler contained a funnel (10.6 cm inner diameter), a rod of 1.5 m PVC pipe (2.5 cm inner diameter), a rubber stopper, two 30‐cm PVC columns (one with an outer diameter of 4.2 cm and the other with an inner diameter of 1.8 cm), and a standard cotton filter. To the inner PVC column, 30 g of ion exchange resin was added (Amberlite MB‐1; Sigma‐Aldrich, Inc., Germany). The IER collectors were positioned at three different locations within each 20 × 20 m plot following the approach of Thimonier (1998), corresponding to a total of 45 IERs. In addition, an IER field blank (IER that was not exposed to water or rain) was installed at each study site by enclosing the column in a plastic container. The samplers were then placed outside the canopy and filled with IER. Over a period of 1 year, IER samples were collected from the resin samplers once every 6 months during the study.

### 2.4 Ion deposition extraction and calculation

A digital balance (JS8001G/A, Mettler‐Toledo Ltd., UK) was used to weigh the ion exchange resins after they had been removed from the IER samplers and dried in an oven at 50°C to a constant weight. Only calcium, Na^+^, Fe^2+^, Cu^2+^, NO_3_^−^, NH_4_^+^ and SO_4_^2−^ were identified in this study. In contrast to NO_3_^−^, NH_4_^+^, and SO_4_^2−^, which are associated with N and S deposition, calcium, Na^+^, Fe^2+^, and Cu^2+^ were selected because they are critical cations for plants in forest ecosystems (Ibrahim et al., 2021; Sheng et al., 2013; Yamashita et al., 2008).

To determine the concentrations of Ca^2+^, Na^+^, Fe^2+^, Cu^2+^, NO_3_^−^, NH_4_^+^, and SO_4_^2−^, 5 g of dry resin was transferred to plastic vials, 40 mL of 1 M KCl was added to the sample, and the sample was shaken vigorously for 30 min. This procedure was repeated to obtain a final volume of 80 mL. Filter paper (Whatman No. 2, Sigma‐Aldrich Inc., USA) was used to filter the samples, and the extracts were made up to 100 mL with deionized water (Ibrahim et al., 2021; Yamashita et al., 2014). A similar procedure was applied to the field blank of the monitoring study.

An atomic absorption spectrophotometer (AAS) (iCE 300, Thermo Fisher Scientific®, NSW, Australia) was used to analyze the samples for the concentrations of Ca^2+^, Na^+^, Fe^2+^, and Cu^2+^. Samples were analyzed for NH_4_^+^ and NO_3_^−^ concentrations using a flow injection analyzer (FIAstarTM5000, FOSS®, Hoganas, Sweden) (Templer et al., 2015), while SO_4_^2−^ was analyzed using an inductively coupled plasma mass spectrometer (5800 ICP‐OES, CA, USA). To ensure that ion concentrations analyzed by AAS (atomic absorption spectroscopy), FIA (flow injection analysis), and ICP (inductively coupled plasma) were validated, we followed standard protocols for calibration with known standards, performed regular quality checks with control samples, used blanks for background correction, and applied rigorous method validation procedures, including assessments of sensitivity, accuracy, and precision. In addition, we used standard reference materials for cross‐validation and adhered to strict maintenance and calibration schedules for the equipment.

The deposition of Ca^2+^, Na^+^, Fe^2+^, Cu^2+^, NO_3_^−^, NH_4_^+^, and SO_4_^2−^ was then calculated using the following formulas developed by Ibrahim et al. (2021):

$$\text{Through}-\text{fall deposition}\;\left({\mathrm{mg}}^{-2}m^{-2}{\text{month}}^{-1}\right)=\frac{\left(\left(\mathrm{Cs}-\mathrm{Cb}\right)\times W\times 10,000\right)}{\left(1000\times \text{funnel area}\right)}$$

where C_s_, concentration per resin sample (mg/kg); C_b_, concentration per resin blank (mg/kg); W, dry weight of the resin (after the sampling period) (30 g); 10,000, conversion factor for funnel area from cm^2^ to m^2^; 1000, conversion factor for resin weight from g to kg; Funnel area, 88.2 cm^2^.

### 2.5 Data and statistical analysis

To determine the differences in the parameters of ion deposition (Ca^2+^, Na^+^, Fe^2+^, Cu^2+^, NO_3_^−^, NH_4_^+^, and SO_4_^2−^), the mean values of these parameters for 5 forest stands and the precipitation time (*n* = 3 plots) were analyzed using the two‐way anova analysis and compared with Duncan's New Multiple Range Test (post‐hoc analysis) at *p* ≤ .05. Comparing ion deposition (Ca^2+^, Na^+^, Fe^2+^, Cu^2+^, NO_3_^−^, NH_4_^+^, and SO_4_^2−^) between the forest stands and the precipitation season within the rehabilitated forests. We applied principal component analysis (PCA) to seven ion variables from three plots and determined the number of principal components (PCs) based on the larger eigenvalues and the explained variance (80%–90% of the total variance). The loadings of each variable on the remaining PCs were analyzed to understand their influence on the variance of the datasets. Assumptions of normality and equal variances were tested, and nutrient deposition variables were log10‐transformed as necessary prior to analysis. All analyses were performed using Statistical Analysis System software, version 9.2 (SAS Corp, 2009) and R version 4.0.5 (R Core Team, USA, 2018).

## 3 RESULTS

### 3.1 Variation in selected ion deposition in the UPMKB rehabilitation forest between five different years of forest stand establishment and precipitation season

The effects of year of forest stand establishment, season, and their interactions were significant for the deposition of Ca^2+^, NO_3_^−^ and SO_4_^2−^, but only the effects of year of forest stand establishment, season, and forest stand‐season interactions were significant for the deposition of Na^+^, Fe^2+^, Cu^2+^ and NH_4_^+^ (Table 2). The results show that the deposition of Ca^2+^ and NH_4_^+^ was consistently lower in the low‐precipitation season than in the high‐precipitation season. We found that there was no obvious pattern for Na^+^, Fe^2+^, Cu^2+^, NO_3_^−^ or SO_4_^2−^ between high‐precipitation and low‐precipitation seasons. The results for each season are then compared with the years of forest stand. The results show that, forest stands from the year 2002, 2005, and 2009 had higher Ca^2+−^, NH_4_^+^ and NO_3_^−^ values in both seasons than those from the year 1996 and 1999. In contrast, higher amounts of Na^+^ and SO_4_^2−^ were measured in forest stands from the low‐precipitation years 2009 and 2002, although the highest value was found in forest stands from the high‐precipitation years 1996.

**TABLE 2:** Variation in the selected chemical depositions of Ca^2+^, Na^+^, Fe^2+^, Cu^2+^, NH_4_^+^, NO_3_^−^ and SO_4_^2−^ via through‐fall that were extracted from ion exchange resin samplers between five different year of forest stand establishments (1996, 1999, 2002, 2005 and 2009) in UPMKB rehabilitation forest (*n* = 3 plots per site).

Forest stand (year)	Precipitation		*F*‐value		
	Low precipitation	High precipitation	Forest stand	Precipitation	Forest stand × precipitation
Ca^2+^ (mg m^−2^ month^−1^)					
1996	202.67 ± 11.80^B,b^	478.00 ± 21.39^A,b^	13.41***	301.08***	7.60***
1999	229.33 ± 21.05^B,b^	490.00 ± 9.24^A,ab^			
2002	266.00 ± 19.43^Bb^	530.00 ± 20.03^A,a^			
2005	355.33 ± 30.42^B,a^	511.33 ± 4.06^B,ab^			
2009	407.33 ± 29.69^B,a^	516.00 ± 6.11^A,ab^			
Na^+^ (mg m^−2^ month^−1^)					
1996	224.02 ± 8.76^A,ab^	251.31 ± 36.83^A,ab^	3.38 ns	5.78 ns	3.04*
1999	200.64 ± 5.03^B,bc^	302.99 ± 11.54^A,a^			
2002	173.96 ± 20.97^A,c^	202.02 ± 30.94^A,b^			
2005	231.30 ± 1.77^A,ab^	258.67 ± 18.74^A,ab^			
2009	259.67 ± 8.25^A,a^	224.63 ± 8.85^B,ab^			
Fe^3+^ (mg m^−2^ month^−1^)					
1996	1.82 ± 0.02^A,a^	1.61 ± 0.05^B,a^	7.62***	9.06**	0.59 ns
1999	1.73 ± 0.04^A,ab^	1.56 ± 0.11^A,ab^			
2002	1.52 ± 0.06^A,c^	1.48 ± 0.06^A,ab^			
2005	1.57 ± 0.08^A,bc^	1.49 ± 0.06^A,ab^			
2009	1.45 ± 0.01^A,c^	1.35 ± 0.04^A,b^			
Cu^2+^ (mg m^−2^ month^−1^)					
1996	0.22 ± 0.009^A,a^	0.23 ± 0.003^A,ab^	3.08*	12.50**	0.60 ns
1999	0.21 ± 0.003^B,a^	0.23 ± 0.003^A,b^			
2002	0.22 ± 0.006^A,a^	0.24 ± 0.009^A,ab^			
2005	0.23 ± 0.01^A,a^	0.24 ± 0.007^A,ab^			
2009	0.23 ± 0.006^A,a^	0.25 ± 0.006^A,a^			
NH_4_^+^ (mg m^−2^ month^−1^)					
1996	1900.7 ± 240.56^B,ab^	3969.5 ± 210.05^A,ab^	0.61 ns	267.07***	4.59**
1999	1429.0 ± 137.51^B,b^	4207.7 ± 420.09^A,ab^			
2002	1667.2 ± 137.51^B,ab^	4048.9 ± 137.51^A,ab^			
2005	1509.6 ± 78.80^B,b^	4525.2 ± 363.81^A,a^			
2009	2047.4 ± 72.49^B,a^	3334.4 ± 137.51^A,b^			
NO_3_^−^ (mg m^−2^ month^−1^)					
1996	206.93 ± 27.00^A,ab^	194.89 ± 33.42^A,bc^	16.05***	11.07*	5.06*
1999	170.50 ± 3.42^A,b^	170.10 ± 5.81^A,c^			
2002	110.46 ± 11.74^B,c^	164.30 ± 3.94^A,c^			
2005	242.01 ± 9.93^A,a^	269.01 ± 47.81^A,b^			
2009	209.15 ± 13.88^B,ab^	371.59 ± 13.96^A,a^			
SO_4_^2−^ (mg m^−2^ month^−1^)					
1996	806.4 ± 98.53^B,ab^	1388.8 ± 63.03^A,a^	3.88*	18.32**	3.85*
1999	678.4 ± 70.18^A,b^	892.5 ± 96.47^A,b^			
2002	1081.60 ± 54.68^A,a^	1040.00 ± 82.27^A,ab^			
2005	696.8 ± 151.23^B,b^	1184.0 ± 150.50^A,ab^			
2009	1065.60 ± 65.35^A,a^	1129.87 ± 75.41^A,ab^			

*Note*: A two‐way anova of the effects of year of forest stand establishment (1996, 1999, 2002, 2005 and 2008) and precipitation (low precipitation and high precipitation) were conducted on Ca^2+^, Na^+^, Fe^3+^, Cu^2+^, NH_4_^+^, NO_3_^−^ and SO_4_^2−^ at the 5% significance level, which were indicated by *p < .05; **p < .01; ***p < .001 and ns = not significant. Means with different lowercase letters within the same row showed differences between forest stands, while means with different uppercase letters within the same column showed differences between precipitation.

### 3.2 Variation in rehabilitated forest selected chemical deposition for low and high precipitation seasons

PCA of seven ion deposition properties for low‐ and high‐precipitation seasons in the UPMKB restoration forest revealed that the first two axes accounted for 88.95% of the total variation in ion deposition concentrations (Table 3). The gradient of decreasing Ca^2+^ and NH_4_^+^ deposition concentrations and increasing iron concentrations is shown on principal component axis 1 (PC1). The principal component axis (PC2) shows a gradient with increasing NO_3_^−^ but decreasing Na^+^ deposition concentrations.

**TABLE 3:** Principal component analysis (PCA) of 7 ion deposition concentrations (Ca^2+^, Na^+^, Fe^*2*+^, Cu^2+^, NH_4_^+^, NO_3_^−^ and SO_4_^2−^) of UPMKB rehabilitated forest for (a) low and high precipitation season and (b) different forest stands.

Parameters	Principal component axis			
	1	2	3	4
(a) Low and high precipitation season				
% Total variation explained	77.84	11.11	7.06	2.48
Cumulative % variation explained	77.84	88.95	96.01	98.49
Loadings of ion exchange resin property				
Ca^2+^	**−0.40**	−0.02	−0.30	−0.37
Na^+^	−0.35	**−0.54**	−0.26	**0.56**
Fe^*2*+^	**0.40**	−0.04	0.34	−0.22
Cu^2+^	−0.36	0.17	**0.71**	**0.41**
NH_4_^+^	**−0.42**	−0.04	−0.01	−0.38
NO_3_^−^	−0.30	**0.78**	−0.20	0.13
SO_4_^2−^	−0.39	−0.24	**0.41**	−0.41
(b) Different forest stands				
% Total variation explained	40.13	15.84	13.57	12.34
Cumulative % variation explained	40.13	55.97	69.54	81.87
Loadings of ion exchange resin property				
Ca^2+^	**0.46**	0.10	−0.05	0.13
Na^+^	−0.00	**0.76**	−0.26	−0.14
Fe^*2*+^	**−0.44**	0.03	**−0.30**	−0.00
Cu^2+^	0.41	−0.11	−0.11	0.18
NH_4_^+^	−0.12	0.34	−0.22	**0.78**
NO_3_^−^	**0.37**	0.29	0.02	−0.23
SO_4_^2−^	0.20	**−0.43**	**−0.64**	0.18

*Note*: Percentage total variation explained by each principal component axis and loadings of each ion exchange resin property for the four principal component axes were presented. Bold values represent moderate and strong loadings of PCA.

**FIGURE 2:**
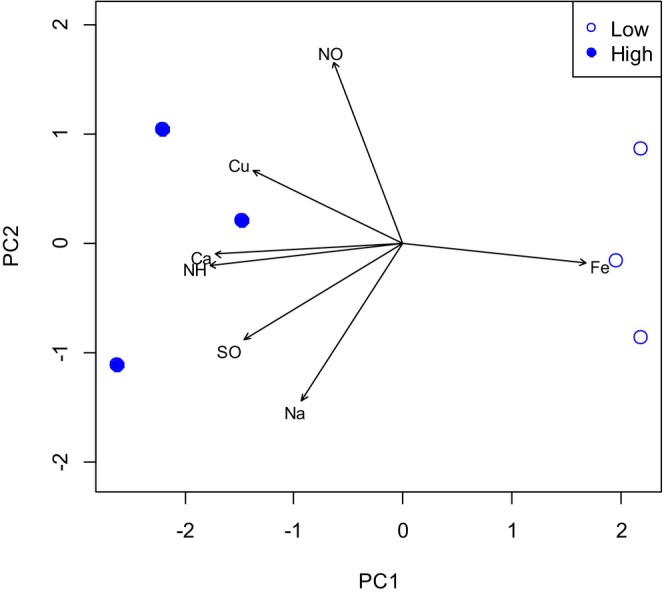
Biplot of principal component (PC) axes 1 and 2 from principal component analysis (PCA) of six ion deposition concentrations of the two precipitation seasons: Low precipitation (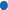) and high precipitation (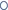) sites (*n* = 3 plots each). Ca, calcium; Na, sodium; Fe, iron; Cu, copper; NH, ammonium; NO, nitrate; SO, sulfate.

**FIGURE 3:**
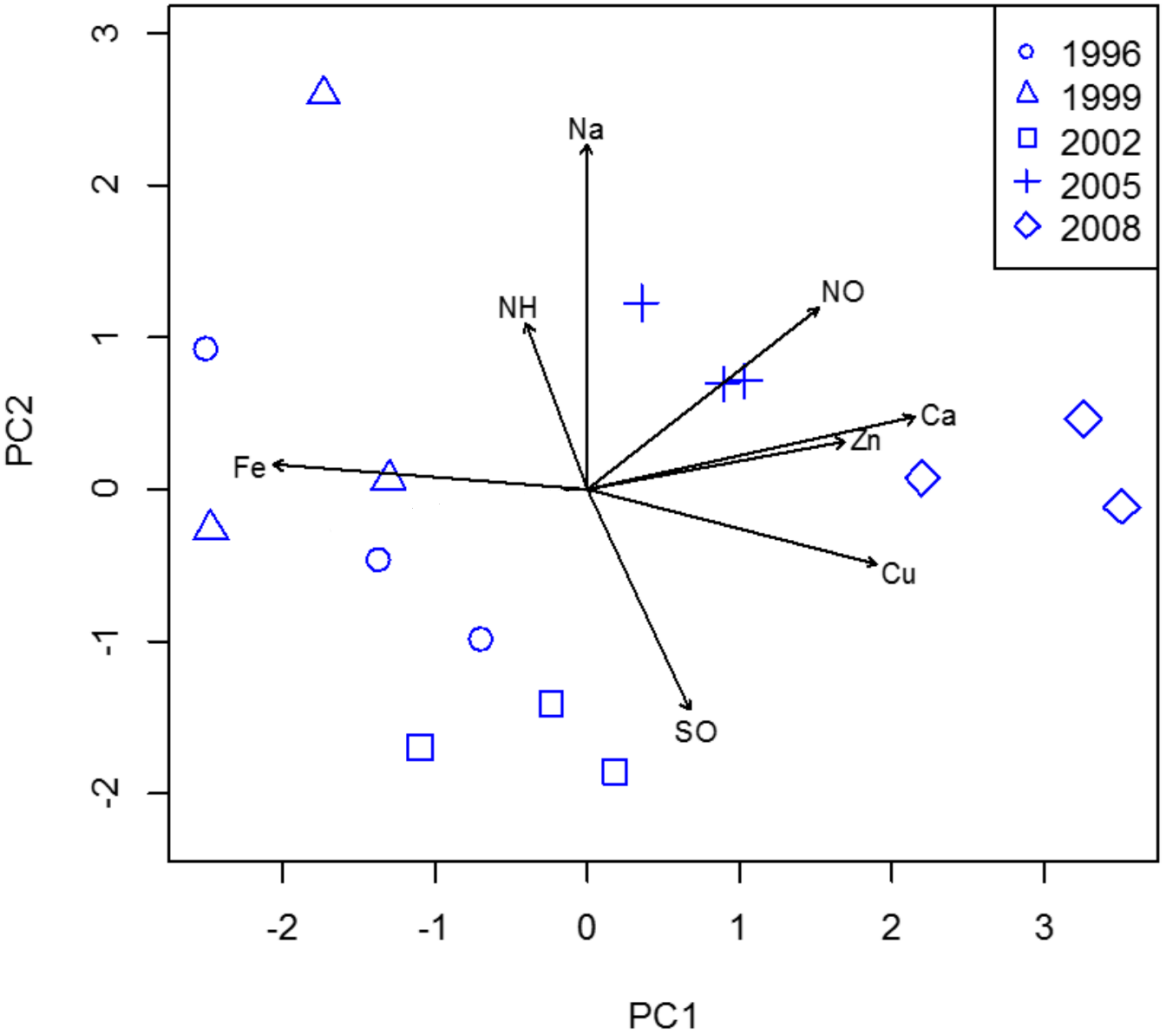
Biplot of principal component (PC) axes 1 and 2 from principal component analysis (PCA) of six ion deposition concentrations of the five forest stands: 1996 (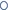), 1999(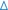) 2002 (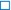), 2005 (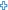), and 2008 (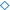) sites (*n* = 3 plots each). Ca, calcium; Na, sodium; Fe, iron; Cu, copper; NH, ammonium; NO, nitrate; SO, sulfate.

The biplot of the PC1 and PC2 axes showed that the low‐precipitation and high‐precipitation seasons were distributed differently in the ordination area (Figure 2). It was found that a high precipitation season is associated with higher concentrations of the ions mentioned: NO_3_^−^, Cu^2+^, Ca^2+^, NH_4_^+^, SO_4_^2−^ and Na^+^. In the biplot, these relationships are represented by a denser grouping (or cluster) of data points on the PC2 axis. As for the low precipitation season, it was likely influenced by an increasing trend in iron (Fe) deposition concentrations in the rehabilitated forest (Figure 3). This could mean that the increasing iron deposition had some influence on the data pattern or distribution in the low precipitation season. This could mean that iron plays an important role in influencing environmental conditions during periods of low precipitation.

### 3.3 Variation in rehabilitated forest selected chemical deposition for different forest stands

Principal component analysis (PCA) of seven ion deposition characteristics for different forest stands in the UPMKB restoration forest showed that the first two axes accounted for 55.97% of the total variation in ion deposition concentrations (Table 3). Principal component axis 1 (PC1) represented a gradient of decreasing iron deposition concentrations accompanied by increasing Ca^2+^ and NO_3_^−^ concentrations. The principal component axis (PC2) represented a gradient of decreasing SO_4_^2−^ deposition concentrations but increasing Na^+^ concentrations.

The dense forest stands (1996, 1999, and 2002) refer to forest areas with a lot of tree cover, and the years mentioned are probably the years in which the data were collected, or the trees were planted (Figure 3). These dense forests were lower on the PC2 axis in the biplot than the open canopy stands (2005 and 2009), suggesting that different environmental conditions prevail in the dense and open canopies. In the case of the dense forests, they were associated with plots that had higher levels of NH_4_^+^, Fe^3+^ and SO_4_^2−^. This could mean that the dense tree cover influences the deposition and/or storage of these chemicals. In contrast, open stands (areas with less tree cover) in the rehabilitated forests were expected to be affected by an increasing trend in concentrations of Na^+^, NO_3_^−^, Ca^2+^ and Cu^2+^ (Figure 3). The expectation expressed here suggests that areas with open canopies may be more susceptible to these chemical depositions due to their increased exposure.

## 4 DISCUSSION

### 4.1 The influence of different precipitation seasons and forest stand years on ion deposition concentrations

The results showed that the concentrations of NO_3_^−^ and SO_4_^2−^ ion deposition interacted in the different years of forest stands and precipitation periods. For the deposition of Na^+^, Fe^2+^, Cu^2+^, and NH_4_^+^, however, no interaction between forest stands and precipitation periods was observed. The study highlights the complex interplay between precipitation, forest stand age and ion deposition and provides important insights into how different ions and forest characteristics influence patterns of ion deposition, that improve forest restoration by informing species selection, nutrient management, and pollution reduction. Adapting restoration strategies to local conditions and anticipating the effects of climate change will lead to more resilient, efficient, and sustainable forest ecosystems and ultimately improve the outcome of forest management and restoration projects.

Our study showed that NH₄^+^ deposition was significantly higher in the low‐precipitation season than in the high‐precipitation season. This discovery is probably due to the volatilization of NH_4_^+^ in the dry season. According to Guo, Hu, and Guan (2022); Guo, Yan, et al. (2022), NH_3_ volatilization from arable soils is positively correlated with total NH₄^+^ deposition in the dry season. This study agrees with the results of Huang et al. (2009), who found that N ion deposition in Guangzhou, southern China, occurred more frequently in spring and summer than in autumn and winter. This is related to the dilution effect of rainwater on precipitation chemistry, with ion concentrations being higher when rainfall is low than when rainfall is high. In the dry season, when there is little rainfall, the suspended matter accumulates, leading to a higher ion deposition, in contrast to the rainy season, when the suspended matter is flushed out with a large amount of rainwater.

In contrast to earlier results on NH₄^+^ deposition, Ca^2+^ deposition was lower during the low‐precipitation season. According to He et al. (2012), a possible explanation for these differences is that Ca^2+^ is precipitated together with Mg^2+^ in older forest stands, such as the forest stands of 1996 and 1999, and this mechanism is important for the regulation and detoxification of these elements in plants, as well as for the protection of plants against herbivory. Magnesium and calcium precipitate in the form of crystals in the leaf tissue of forest leaves, which leads to a lower Ca^2+^ content in older forest stands (see Table 2).

Regarding the results of forest stands for each season, we found that in both seasons younger forest stands (2002, 2005, and 2009) had a greater impact on Ca^2+^, NH_4_^+^, and NO_3_ values than older forest stands (1996 and 1999). We hypothesize that the differences in canopy cover between older and younger forest stands could be a cause of these fluctuations. Canopy structure, such as canopy cover and leaf area index, may influence ion deposition via through‐fall (Ibrahim et al., 2021). Older forest stands (1996 and 1999) with canopy openness of 56%–61% and 77%–79%, respectively (see Heng et al., 2017), have greater interception capacity between canopies, which has a small effect on ion deposition in the throughfall. Several studies have found that a dense canopy structure (Crockford & Richardson, 2000; Park & Cameron, 2008) and high leaf area index (Whelan et al., 1998) can facilitate lower ion deposition in the throughfall.

### 4.2 The effects of a single precipitation season and forest stand year on ion deposition concentrations

We applied PCA analysis to determine the effects of precipitation season and year of forest cover on ions deposition concentrations, as two‐way anova could not clearly identify their contributions. The initial PCA results were conducted between low‐precipitation and high‐precipitation seasons regardless of the year of forest cover, and the PCA results in Table 3 confirmed that Ca^2+^ and NH_4_^+^ deposition tended to decrease in the high precipitation season. We also found that more ions were deposited in seasons with high precipitation, especially Ca^2+^, Na^+^, NH_4_^+^, Cu^2+^, NO_3_^−^ and SO_4_^2−^, while only Fe^2+^ was deposited in seasons with low precipitation. The result was explained by the statics of iron uptake by plants, where iron in older leaves is not transferred to young leaves (Zandalinas et al., 2019). In view of this, iron deposition during high rainfall could be minimized by ensuring a constant supply of iron for their growth.

Regardless of precipitation time, the second PCA results between different forest stands (1996, 1999, 2002, 2005, and 2009) showed that Ca^2+−^ and NO_3_^−^ deposition tended to increase, while Na^+−^ and SO_4_^2−^ deposition tended to decrease in younger forest stands (2002, 2005, and 2009). Previous research has shown that seasonal precipitation patterns can influence community productivity in different ecosystems, with wetness determined by climatological anomalous accumulation (CAA) being more spatially uniform than potential evapotranspiration (PET) in Amazonian forests (Luo, 2024). In addition, the interactions between atmospheric deposition and forest canopy were observed to vary with season, with the deposition of certain ions by precipitation during the dormant season is significantly higher compared to wet precipitation under both deciduous and mixed coniferous forests (Houle et al., 1999). Additionally, the impact of precipitation on stand transpiration has been highlighted, with reports suggesting that early growth season precipitation positively influences stand transpiration due to soil water recovery following dry periods (Yan et al., 2016). Moreover, the relationship between precipitation and forest structural complexity has been emphasized, indicating that dry periods can pose a threat to the complexity of the understory layer.

### 4.3 Management implications of this study on precipitation season and forest stands

The results of the present study can significantly improve forest restoration processes by providing a detailed understanding of ion deposition patterns in rehabilitated forests. By analyzing how different ions such as Ca^2+^, Na^+^, Fe^2+^, Cu^2+^, NH₄^+^, NO_3_^−^, and SO_4_^2−^ are deposited in forest stands of different ages and during different precipitation seasons, this study provides valuable insights that can guide the selection of appropriate tree species and forest management practices. Understanding these patterns enables the optimization of nutrient cycling and soil fertility, which are crucial for the health and growth of rehabilitated forests.

Based on the results of the study, which emphasizes the significant influence of precipitation seasonality and the maturity of forests on ions deposition, customized management methods can be developed. For example, younger forest stands may require additional nutrients during low rainfall seasons to ensure their optimal growth and health. This approach ensures that forest restoration projects are more effective and efficient, as they can consider the specific needs of different forest stands based on their age and prevailing climatic conditions.

The study also emphasizes the crucial role of seasonal precipitation in the nutrient cycle. By planning measures that improve nutrient availability during dry seasons, forest managers can support the overall health and resilience of the ecosystem. This knowledge is particularly valuable for maintaining the balance of essential nutrients in the soil, and thus for robust plant growth and ecosystem sustainability.

Moreover, the interaction between forest canopy and ion deposition has implications for pollution reduction. The results of the study can guide the design of restoration projects that not only focus on forest health but also contribute to improving air quality. Selecting tree species with canopies that effectively intercept and mitigate pollutants in the atmosphere can help reduce the negative effects of acid rain and other forms of pollution, thus increasing the ecological benefits of forest restoration.

The long‐term sustainability of forest restoration efforts can also be supported by the knowledge gained in this study. By understanding the deposition and cycling of essential ions, forest managers can develop sustainable management practices that adapt to climate variability and anthropogenic impacts. Ongoing monitoring and assessment of ion deposition and forest health will enable the implementation of flexible management plans that respond to changing environmental conditions and ensure the resilience and longevity of rehabilitated forests.

## 5 CONCLUSIONS

In this study, the effects of precipitation time and forest cover on the deposition of certain ions in the restored forest were thoroughly investigated. Contrasting patterns in the deposition of Ca^2+^, Na^+^, Fe^2+^, Cu^2+^, NO_3_^−^, NH_4_^+^, and SO_4_^2−^ ions over precipitation indicate that precipitation time and forest cover influence ion deposition in rehabilitated forests. These results are important because the precipitation time and forest cover determine the availability of nutrients. This disrupts the closed nutrient cycles of these forests and further alters ecosystem functioning and biodiversity in these increasingly rare natural habitats. The results of this study set the stage for future studies that will require a coordinated series of longer‐term, studies at the soil‐ and water‐level studies to fully assess the detrimental effects of rainfall timing and forest stands on the overall ecosystem function and biodiversity of these forests. These results are crucial as they relate to climate change projections that assume more intense and frequent hydrological events and can inform ecological restoration processes to improve forest resilience.

## FUNDING INFORMATION

This study was supported by the Malaysian Ministry of Higher Education through the Fundamental Research grant scheme (FRGS: 5523701), Universiti Putra Malaysia through the Research University grant scheme (RUGS: 9199765), the Japanese Ministry of the Environment through the Environmental Research and Technology Development Fund (B‐0801), and the Mitsubishi Corporation Trust Fund (6380500).

## CONFLICT OF INTEREST STATEMENT

The author declares that there is no conflict of interest.

## Data Availability

Research data are not shared.

## References

[pei370005-bib-0046] Cao, M. , Zou, X. , Warren, M. , & Zhu, H. (2006). Tropical forests of Xishuangbanna, China. Biotropica, 38(3), 306–309.

[pei370005-bib-0002] Crockford, R. H. , & Richardson, D. P. (2000). Partitioning of rainfall into through‐fall, stemflow, and interception: Effect of forest type, ground cover, and climate. Hydrological Processes, 14, 2903–2920.

[pei370005-bib-0004] EANET (Acid Deposition Monitoring Network in East Asia) . (2021a). The fourth periodic report of the state of acid deposition in East Asia. Network Center for EANET, Asia Center for Air Pollution Research.

[pei370005-bib-0005] EANET (Acid Deposition Monitoring Network in East Asia) . (2021b). Data report 2020. Network Center for EANET, Asia Center for Air Pollution Research.

[pei370005-bib-0006] Gessler, A. , Rienks, M. , & Rennenberg, H. (2002). Stomatal uptake and cuticular adsorption contribute to dry deposition of NH3 and NO2 to needles of adult spruce (*Picea abies*) trees. New Phytologist, 156, 179–194. 33873281 10.1046/j.1469-8137.2002.00509.x

[pei370005-bib-0007] Guo, J. , Hu, S. , & Guan, Y. (2022). Regime shifts of the wet and dry seasons in the tropics under global warming. Environmental Research Letters, 17(10), 104028.

[pei370005-bib-0008] Guo, S. , Yan, T. , Zhai, L. , Yen, H. , Liu, J. , Li, W. , & Liu, H. (2022). Nitrogen transport/deposition from paddy ecosystem and potential pollution risk period in Southwest China. Watermark, 14(4), 539.

[pei370005-bib-0009] Hambuckers, A. , & Remacle, J. (1993). Relative importance of factors controlling the leaching and uptake of inorganic ions in the canopy of a spruce forest. Biogeochemistry, 23(2), 99–117.

[pei370005-bib-0010] Hämmerle, A. I. , Wessely, J. , Baatar, U. O. , Essl, F. , Moser, D. , Jiménez‐Alfaro, B. , Jandt, U. , Agrillo, E. , Stančić, Z. , Dirnböck, T. , & Dullinger, S. (2018). A new method for jointly assessing effects of climate change and nitrogen deposition on habitats. Biological Conservation, 228, 52–61.

[pei370005-bib-0011] He, H. , Bleby, T. M. , Veneklaas, E. J. , Lambers, H. , & Kuo, J. (2012). Precipitation of calcium, magnesium, strontium and barium in tissues of four Acacia species (Leguminosae: Mimosoideae). PLoS One, 7(7), e41563. 22848528 10.1371/journal.pone.0041563 PMC3405136

[pei370005-bib-0012] Heng, K. , Nik, M. , Seca, G. , Osumanu, H. A. , Jemat, S. , Kin, K. , & Kiong, J. T. C. (2011). Forest structure assessment of a rehabilitated forest. American Journal of Agricultural and Biological Sciences, 6(2), 256–260.

[pei370005-bib-0013] Heng, R. K. J. , Majid, N. M. A. , Gandaseca, S. , Ahmed, O. H. , Jemat, S. , Kin, M. K. K. , & Kiong, J. T. C. (2017). Evaluation of rehabilitated forest stands development using hemispheric photograph. Journal of Advanced Research Design, 36(1), 25–33.

[pei370005-bib-0014] Hicks, B. B. , Saylor, R. D. , & Baker, B. D. (2016). Dry deposition of particles to canopies–a look back and the road forward. Journal of Geophysical Research: Atmospheres, 121(24), 14–691.

[pei370005-bib-0015] Houle, D. , Ouimet, R. , Paquin, R. , & Laflamme, J. (1999). Interactions of atmospheric deposition with a mixed hardwood and a coniferous forest canopy at the Lake Clair watershed (Duchesnay, Quebec). Canadian Journal of Forest Research, 29(12), 1944–1957.

[pei370005-bib-0016] Huang, D. Y. , Xu, Y. G. , Zhang, H. H. , & Lan, J. B. (2009). Chemical composition and seasonal variation of acid deposition in Guangzhou, South China: Comparison with precipitation in other major Chinese cities. Environmental Pollution, 157(1), 35–41. 18801606 10.1016/j.envpol.2008.08.001

[pei370005-bib-0018] Ibrahim, M. H. , Metali, F. , U Tennakoon , K., & Sukri, R. S. (2021). Impacts of invasive Acacias on ion deposition in a coastal Bornean tropical heath forest. Journal of Forest Research, 27, 20–27.

[pei370005-bib-0019] Igawa, M. , Matsumura, K. , & Okochi, H. (2002). High frequency and large deposition of acid fog on high elevation forest. Environmental Science & Technology, 36(1), 1–6. 11811474 10.1021/es0105358

[pei370005-bib-0020] Khormali, F. , Ajami, M. , Ayoubi, S. , Srinivasarao, C. , & Wani, S. P. (2009). Role of deforestation and hillslope position on soil quality attributes of loess‐derived soils in Golestan province, Iran. Agriculture, Ecosystems & Environment, 134, 178–189.

[pei370005-bib-0021] Liu, G. , Cai, Y. , Kalla, P. , Scheidt, D. , Richards, J. H. , Scinto, L. J. , Gaiser, E. , & Appleby, C. (2008). Mercury mass budget estimates and cycling seasonality in the Florida everglades. Environmental Science & Technology, 42(6), 1954–1960. 18409620 10.1021/es7022994

[pei370005-bib-0022] Luo, L. (2024). Climate seasonality of tropical evergreen forest region. Watermark, 16(5), 749.

[pei370005-bib-4000] Malaysian Meteorological Department (MMD) . (2011). Annual report on rainwater patterns and distribution in malaysia. Malaysian Meteorological Department, Ministry of Science, Technology and Innovation.

[pei370005-bib-0025] Mladenov, N. , Williams, M. W. , Schmidt, S. K. , & Cawley, K. (2012). Atmospheric deposition as a source of carbon and nutrients to an alpine catchment of the Colorado Rocky Mountains. Biogeosciences, 9(8), 3337–3355.

[pei370005-bib-0027] Park, A. , & Cameron, J. L. (2008). The influence of canopy traits on through‐fall and stemflow in five tropical trees growing in a Panamanian plantation. Forest Ecology and Management, 255, 1915–1925.

[pei370005-bib-0029] Qian, W. , & Tang, S. (2010). Identifying global monsoon troughs and global atmospheric centers of action on a pentad scale. Atmospheric and Oceanic Science Letters, 3(1), 1–6.

[pei370005-bib-0045] Rummel, U. , Ammann, C. , Kirkman, G. A. , Moura, M. A. L. , Foken, T. , Andreae, M. O. , & Meixner, F. X. (2007). Seasonal variation of ozone deposition to a tropical rain forest in southwest Amazonia. Atmospheric Chemistry and Physics, 7(20), 5415–5435.

[pei370005-bib-0031] Sase, H. , Takahashi, A. , Sato, M. , Kobayashi, H. , Nakata, M. , & Totsuka, T. (2008). Seasonal variation in the atmospheric deposition of inorganic constituents and canopy interactions in a Japanese cedar forest. Environmental Pollution, 152(1), 1–10. 17658672 10.1016/j.envpol.2007.06.023

[pei370005-bib-0048] Sheng, W. , Yu, G. , Jiang, C. , Yan, J. , Liu, Y. , Wang, S. , Wang, B. , Zhang, J. , Wang, C. , Zhou, M. , & Jia, B. (2013). Monitoring nitrogen deposition in typical forest ecosystems along a large transect in China. Environmental Monitoring and Assessment, 185(1), 833–844. 22411032 10.1007/s10661-012-2594-0

[pei370005-bib-0033] Statistical Analysis System (SAS) . (2009). SAS/STAT software (Version 9.2) [Statistical software]. https://www.sas.com/en_us/software/stat.html

[pei370005-bib-0034] Talkner, U. , Krämer, I. , Hölscher, D. , & Beese, F. O. (2010). Deposition and canopy exchange processes in central‐German beech forests differing in tree species diversity. Plant and Soil, 336(1), 405–420.

[pei370005-bib-0035] Tan, S. , Zhao, H. , Yang, W. , Tan, B. , Ni, X. , Zhang, Y. , & Wu, F. (2018). The effect of canopy exchange on input of base cations in a subalpine spruce plantation during the growth season. Scientific Reports, 8(1), 9373. 29921971 10.1038/s41598-018-27675-9 PMC6008401

[pei370005-bib-0036] Templer, P. H. , Weathers, K. C. , Lindsey, A. , Lenoir, K. , & Scott, L. (2015). Atmospheric inputs and nitrogen saturation status in and adjacent to class I wilderness areas of the northeastern US. Oecologia, 177(1), 5–15. 25407620 10.1007/s00442-014-3121-5

[pei370005-bib-0047] Thimonier, A. (1998). Measurement of atmospheric deposition under forest canopies: Some recommendations for equipment and sampling design. Environmental Monitoring and Assessment, 52(3), 353–387.

[pei370005-bib-0037] United States Environmental Protection Agency (US‐EPA) . (2005). Report of acid rain program progress 2004. U.S. Environmental Protection Agency (EPA).

[pei370005-bib-0038] Xu, H. , Lian, X. , Slette, I. J. , Yang, H. , Zhang, Y. , Chen, A. , & Piao, S. (2022). Rising ecosystem water demand exacerbates the lengthening of tropical dry seasons. Nature Communications, 13(1), 4093. 10.1038/s41467-022-31826-y PMC9283447 35835788

[pei370005-bib-0039] Yamashita, N. , Ohta, S. , & Hardjono, A. (2008). Soil changes induced by Acacia mangium plantation establishment: Comparison with secondary forest and Imperata cylindrica grassland soils in South Sumatra, Indonesia. Forest Ecology and Management, 254, 362–370.

[pei370005-bib-0041] Yamashita, N. , Sase, H. , & Kobayashi, R. (2014). Atmospheric deposition versus rock weathering in the control of streamwater chemistry in a tropical rain‐forest catchment in Malaysian Borneo. Journal of Tropical Ecology, 30, 481–492.

[pei370005-bib-0042] Yan, M. , Zhang, J. , He, Q. , Shi, W. , Otsuki, K. , Yamanaka, N. , & Du, S. (2016). Sapflow‐based stand transpiration in a semiarid natural oak forest on China's Loess Plateau. Forests, 7(12), 227.

[pei370005-bib-0043] Zandalinas, S. I. , Song, L. , Sengupta, S. , McInturf, S. A. , Grant, D. G. , Marjault, H. , Castro‐Guerrero, N. A. , Burks, D. , Azad, R. K. , Mendoza‐Cozatl, D. G. , Nechushtai, R. , & Mittler, R. (2019). Expression of a dominant‐negative atneet‐h89c protein disrupts iron–sulfur metabolism and iron homeostasis in arabidopsis. The Plant Journal, 101(5), 1152–1169. 31642128 10.1111/tpj.14581

[pei370005-bib-0044] Zhang, G. , Zhang, J. , & Liu, S. (2007). Characterization of nutrients in the atmospheric wet and dry deposition observed at the two monitoring sites over Yellow Sea and East China Sea. Journal of Atmospheric Chemistry, 57(1), 41–57.

